# Enabling Biomimetic Place-Based Design at Scale

**DOI:** 10.3390/biomimetics5020021

**Published:** 2020-05-18

**Authors:** Samantha Hayes, Jane Toner, Cheryl Desha, Mark Gibbs

**Affiliations:** 1 Cities Research Institute, Griffith University, Brisbane 4111, Australia; c.desha@griffith.edu.au; 2 Biomimicry Australia, Melbourne 3000, Australia; jtoner8@gmail.com; 3 Institute for Environments, Queensland University of Technology, Brisbane 4000, Australia; mt.gibbs@qut.edu.au

**Keywords:** biomimicry, built environment, design, place-based design, regenerative, urban development

## Abstract

Amidst the inter-related challenges of climate change, resource scarcity, and population growth, the built environment must be designed in a way that recognises its role in shaping and being shaped by complex social and ecological systems. This includes avoiding the degradation of living systems in the design and construction of buildings and infrastructure, as well as enhancing the built environment’s resilience to disturbance by those systems. This paper explores the potential for biomimetic place-based design (BPD) to inform resilient and regenerative built environment outcomes by learning from local ecosystems. One recognised hurdle is the upfront resourcing required to establish the biomimetic knowledge base for each project. However, conducting BPD projects at-scale (i.e., city or region) can improve the method’s value-proposition by better leveraging upfront research efforts, design concepts, and strategies. This research identifies existing barriers to the widespread adoption of BPD and presents an action framework for capability-building across industry, government, and academia to enable application at-scale. Drawing on findings from workshops in the USA and Australia, it creates a resource for colleagues looking to apply BPD in a city or region and offers next steps for research and development.

## 1. Introduction

Cities are impacted by a range of complex and dynamic stressors, including changing climate conditions and increased intensity of extreme weather events, along with the interplay of trends such as population growth, biodiversity loss, and resource scarcity [1,2]. Designing for complex and unpredictable challenges can be difficult. However, while increasing in scale and complexity, these challenges are not entirely new. Plants and animals within ecosystems have been responding and adapting to conditions such as extreme heat, flooding, materials scarcity, and over-competition in various forms for approximately 3.8 billion years [3,4].

Local ecosystems provide fertile ground for locally-optimised design strategies. Peters, Lazarus and Crawford, and Taylor Buck [5,6,7] refer to the significant opportunity to investigate biological strategies developed by ‘champion adapters’ within ecosystems and translate these into design principles for built environment design and engineering. Such approaches support a design that is locally-attuned, adaptable, and resilient to local operating conditions and challenges [7,8]. Biomimetic place-based design (BPD) is one such approach that seeks to leverage these biological learnings for application in the built environment. It includes the Genius of Place (GoP) design methodology developed by Baumeister et al. [8], which uses eight steps to guide designers in identifying functional challenges, discovering organisms that are well-adapted to delivering that function or managing that challenge, and translating these to design concepts that are appropriate for the context. 

The GoP design methodology offers two approaches for analysis—‘biology to design’, and ‘challenge to biology’. In the former, biologists investigate a selected ecosystem and identify species that have developed unique adaptations to local operating conditions and challenges. Biological strategies and mechanisms are abstracted into design principles used to inform design strategies well-suited for that place. In the latter, the approach is to begin by identifying a functional challenge, and then searching specifically for local organisms that have adapted successfully to that challenge.

Where GoP has been established as a detailed biomimetic design methodology, here we introduce ‘biomimetic place-based design’ as an overarching descriptor that captures not only GoP, but all (existing and emerging) biomimetic design approaches and frameworks that draw on local and ‘place-based’ ecological design strategies. Recognising GoP as a foundational methodology in this space, an increasing focus on biomimetic design means that it is likely that supplementary and alternative design methodologies will continue to emerge over time, each using biomimicry to inform place-based design. Emerging methodologies may expand on selected elements of the design process or biomimicry ethos, including, for example, direct connection to nature through the incorporation of biophilic design. BPD allows these aligned and adjacent methodologies to also be captured. The learnings and lessons outlined below are applicable not only to projects using the GoP approach but presumably also to other divergent frameworks operating in the same sphere. As such, ‘BPD’ seeks to capture any design methodology that satisfies the criteria of being both place-based, and biomimetic in nature. 

This paper explores the opportunity for biomimetic place-based design at-scale, leveraging the GoP methodology to offer a higher-level perspective of the resources and capabilities required to implement this design approach for cities and regions. The BPD approach to generating design concepts requires expert research and translation across disciplines, with involvement from biologists, designers, engineers, and other industry, government, and academia partners. Early piloting of biomimetic design has identified the time and resource-intensive ‘upfront research burden’ as a key challenge and barrier to broader uptake [9]. As such, a recently emerging practice is to conduct this type of study for a region or ecosystem type, rather than an individual project site. Once the biological research has been undertaken for a particular organism, and design principles have been established, they can be relevant not only for the site under investigation but for many geographic areas or ecosystem types with similar functional design challenges. This can allow for an improved value-proposition for the upfront knowledge-gathering effort, with broader utilisation of the research findings and subsequent design concepts and strategies, across multiple built environment projects.

The authors were interested in exploring the challenges and barriers to implementing the BPD approach at-scale. In this paper, priority action areas are identified for industry, government, and academia in order to advance biomimetic place-based design approaches in cities and regions through education, training, and resource development. The resulting capability building framework identifies where practitioners can leverage existing knowledge, as well as opportunities to further build capability across sectors. The paper concludes with opportunities for further investigation and application.

## 2. Design Approaches for Complex Systems

As the built environment impacts on socio-ecological systems, it is also—in turn—impacted by them [1,10]. With increasing awareness of the complexity of these interrelationships and feedback loops, opportunities are arising for more dynamic and innovative approaches to design, construction, and operation. Such approaches require a move beyond siloed and discrete technical efforts to more holistic design methodologies that account for the complexity and the dynamics of changing local conditions [11,12,13]. 

For built environment design, this requires an appreciation of the many interconnections and interdependencies between built assets and their social, cultural, and ecological contexts, through a renewed focus on ‘designing for place’ [14]. It is increasingly impractical to perceive built environment assets as discrete technical entities without appreciating their interactions with surrounding assets and the environment. Indeed, buildings and infrastructure exist as integrated elements of complex socio-eco-technological systems [15,16]. Researchers such as Hes and Du Plessis, and Chester and Allenby [11,17] advocate for design to strive for adaptability and the capacity to respond to complexity, uncertainty, and unpredictability. It should be place-based, locally-attuned, and responsive [14]. Further, it should seek not only to recognise these socio-ecological systems but also to actively regenerate them [18]. These concepts form key tenants of the emerging discipline of ‘regenerative design’ in the built environment.

Regenerative design seeks to build, rather than reduce, social and natural capital in place [19,20]. Where ‘green’ building frameworks look to improve efficiencies and minimise emissions, for example, and ‘sustainability’ rating schemes broaden this to cover economic and human criteria (See for example [21,22,23,24,25]), regenerative design moves further again along this spectrum [17,26,27,28]. It has been argued that green building and infrastructure frameworks remain largely mechanistic approaches to damage reduction, failing to account for the complex and dynamic natures of the socio-ecological systems within which those built environment assets exist [18,29]. Regenerative design is positioned as an alternative integrated approach that incorporates both these individual technical approaches and a whole living systems approach to a design that engages with socio-ecological systems for ‘adaptation, resilience and regeneration’ [18,29].

### 2.1. Biomimicry as an Approach to Regenerative Place-Based Design

Biomimicry encapsulates many of these ideas and offers design methodologies to translate them from theory to practice. Biomimetic design principles recognise regeneration and self-repair, for example, as a fundamental design characteristic in natural systems. At the system level, biomimicry recognises the interconnected and complex relationships between the individual and broader socio-ecological systems—a perspective central to regenerative design philosophies, and one that shifts thinking beyond narrow damage control. Concepts of net positive or ‘generous’ design, as well as symbiosis, are prominent in nature and in biomimetic design. By drawing directly on living systems for design inspiration, it is possible to tap into these tried and tested examples of adaptability, resilience, multifunctionality, and regeneration. As such, biomimicry offers a valuable enabler for actioning regenerative design ideas.

Biomimetic place-based design (BPD) views local ecosystems as rich sources of information and inspiration for solutions that are well-adapted to the functional challenges of their places. For example, in a location subject to flooding from extreme rainfall events, some local plants will have optimized strategies for the functional challenge of diffusing water. By identifying these plants and determining the strategies and mechanisms for water diffusion, design principles can be developed for designers to emulate. Created and piloted by Baumeister et al. [8] at Biomimicry 3.8, the ‘Genius of Place’ (GoP) framework was developed to capture the process and methodology for drawing on local biological design strategies to inform design and engineering solutions [8]. It has similarities to the Genius Loci concept adopted in built environment disciplines to describe a sense of place or, the ‘spirit of a place’ [30,31,32], however, it shifts the concept from human-centric to eco-centric by using targeted methodologies for extracting specific design strategies from plants, animals, and ecosystems to inform management of context-specific challenges and operating conditions. Using this design methodology, it is possible to identify place-based design strategies that address key operating conditions and challenges. Several public reports document early efforts by public and private sector organisations to implement the GoP design methodology in response to local challenges (See for example [6,33,34,35]).

### 2.2. Advancing Biomimetic Place-Based Design At-Scale

Several tools, resources, and education programs are now emerging to support the practice of this design approach. However, the upfront research burden remains high, particularly given limitations in eco-literacy within built environment disciplines. Recognising the extent of research required in order to identify relevant organisms, investigate biological strategies, and translate these into usable design strategies, it is likely that such efforts will continue to be too onerous for many individual built environment projects, not aligning well with the condensed timeframes of project design and construction. Considering ways to improve the method’s value-proposition, we now explore an opportunity to conduct biomimetic place-based design projects at-scale, moving beyond the scope of a single site to investigate an entire ecosystem type, settlement, or city. Options include investigation at the biome level (for example, forest, desert, tundra, grassland), or more targeted ecosystem assessments (for example, mangrove forest). Project teams could investigate one ecosystem type, or analyse several within a study area. Within one site or city, for example, there are likely to be a mosaic of unique ecosystem types that have evolved in response to local conditions, climate, geography, and a host of other relevant context-specific parameters. By adopting a city-wide approach, it is possible to commence with one subset of the living system, and progressively expand the breadth and depth of investigation over time.

One early example of this approach can be seen in the work of a global design firm that identified that many of their international projects occurred within a single biome type, and that they were encountering several common challenges across these sites. After shortlisting key challenge areas for further investigation, they researched organisms that were well adapted to those challenges within the identified biome type and translated the biological strategies into usable design concepts. The resulting publication has since been used to inspire design approaches for several built environment projects. Following on from this, the firm has recently conducted a second similar project investigating biomimetic place-based design strategies for a single geographical region. This approach has allowed for the learnings to be leveraged across multiple projects, while also reducing the time and resource burden at the project level.

Moving forward, there is an opportunity to mirror this type of approach with BPD projects that research and develop design strategies appropriate for adoption in other biomes, regions, or cities. While the above examples have been led by the private sector, there are opportunities for government, industry, and academia to each contribute moving forward, with interdisciplinary collaboration integral to successful piloting and adoption. 

### 2.3. Innovation Diffusion in Complex Systems

To support and enable uptake of innovative biomimetic design approaches, it is helpful to turn to existing knowledge on innovation adoption in complex systems. According to the innovation diffusion theory developed by Nan et al. [36], there are many attributes that contribute to innovation diffusion in such systems, including the role of adopters and influencers, their interactions, and the external environment in which they operate. They note that, though a particular innovation may offer much promise, there is no guarantee of widespread uptake, recognising that agents within the system must have awareness, motivation, and capability in order to adopt an innovation and contribute to its diffusion. Innovations that are more radical; more complex; less easy to use; less compatible with adopters’ contexts, cognitions, and behaviours; less trialable; and less observable are typically considered more arduous to adopt [36], reducing the likelihood and scale of adoption within a system. The BPD approach and GoP methodology satisfy several of these criteria, including the potential complexity, level of work associated with building foundational knowledge, limited number of previous pilot projects, and lack of visibility. According to Nan et al. [36], when arduousness is high, the capability of the adopters and influencers in the system must also be high. Capability in this context relates to the level of understanding and capacity for agents to implement an innovation and the availability of resources required to do so. In summary, increasing capability reduces the overall arduousness of implementing the innovation, and provides the necessary content for individuals within the network to effectively communicate the innovation and promote adoption. Capability-building involves a focus on skill development, education, and training. The term is used intentionally here as distinct from capacity building, which prioritises workforce development and organisational capacity [37]. Using this lens of innovation diffusion, we identify key existing barriers to the implementation of BPD at-scale by industry and government. Finding that many of these barriers relate to education and knowledge gaps, we develop and present an action framework for building BPD capability across sectors.

## 3. Method: Workshops

This investigation used three workshops, conducted with academic and industry participants in the USA and Australia, to explore opportunities for implementing and mainstreaming BPD.

### 3.1. Workshops: Scoping and Industry Engagement

Research workshops enable participants to engage in a way that allows group dynamics and interactions to advance discussion beyond that of individual exchanges [38,39]. The comments, enquiries, or perspectives of one participant may prompt a line of thought for other participants that would not have otherwise emerged, a benefit that is particularly relevant in an emerging area of study, where participants may not have extensive experience of familiarity with the topic under discussion. 

To support this interaction and engagement, attendees completed activities in small groups within the larger workshop group, with opportunities to provide feedback and emergent concepts to the workshop facilitator. Workshop findings consisted of the documented responses to the activities undertaken by attendees, and inductive analysis was used to identify emergent themes and priority areas. 

Three workshops were conducted, discussing the opportunity for further refinement and uptake of the BPD approach, including key actions for government, academia, and industry. Meta-data relating to the workshops are provided in Table 1.

The first workshop (WS1), conducted in the USA (Phoenix), was used to highlight major gaps and opportunities for further consideration. The workshop was held in Phoenix, the location of The Biomimicry Center at Arizona State University, where (in collaboration with Biomimicry 3.8) the Genius of Place framework has been developed and championed, and where a Master’s of Science (Biomimicry) is offered. Arizona State University also has a School of Sustainable Engineering and the Built Environment, as well as The Design School, making it well-placed for expertise across these three disciplines. The authors targeted academic specialists within the fields of sustainability, engineering, biomimicry, and design. It was conducted as a preliminary scoping workshop to highlight any major challenges, uncertainties, and opportunities in the application of BPD. 

The second (WS2) and third (WS3) workshops, conducted in Australia (Brisbane and Sydney), were targeted primarily at industry participants and were designed to identify priorities, action areas and responsibilities to support broader industry piloting and development. With the author team being based in Australia, Brisbane and Sydney were selected to provide multiple perspectives on both the level of opportunity and the existing capability in Australian cities. Participants were practitioners involved in infrastructure sustainability, project delivery, design, and engineering. Participants for all workshops were identified through publication searches and chain-referral or ‘snowball’ sampling, where participants were asked to suggest others within their field who may offer valuable insights. For the Australian workshops, an invitation was also published by the lead author on LinkedIn, in order to draw on industry networks in the field. 

### 3.2. Workshop Format

Each workshop was approximately 2.5 h in duration and conducted in-person, with outputs captured as written responses to pre-prepared workshop activities. There were 15 attendees at the Phoenix workshop (WS1), nine attendees at the Brisbane workshop (WS2), and 15 at the Sydney workshop (WS3). The workshops were conducted as structured sessions with the following agenda:

- Research Context (WS1–Phoenix only)
- Overview of the current status of biomimetic engineering (research and application) in the built environment (all workshops);
- Introduction to three system-level biomimicry frameworks (all workshops); and
- Small group discussions and structured activities exploring opportunities and challenges for uptake of the three frameworks (all workshops).

BPD, specifically the GoP design methodology, was introduced to all participants at the commencement of each workshop, with reference to the four key phases of GoP: scoping, discovering, creating, and evaluating. In the scoping workshop (WS1), participants formed three groups and were asked to discuss and record the key opportunities and challenges that emerged from their introduction to and discussion of the potential of the Genius of place design methodology. 

In the Industry workshops, participants worked in small groups to explore opportunities for implementation of the Genius of Place or BPD approach in their city. Building on the findings of the scoping workshop, introductory content provided to participants before the activity was expanded to highlight potential key challenges and opportunities. Recognising the industry audience and looking to the future application and advancement of BPD, participants in these workshops were asked to focus on key *actions* required to support further uptake, with specific consideration of how this could be advanced within their city. To support the generation of tangible actions, the activity asked participants to consider actions across four key phases: (1) biological research; (2) design development; (3) develop guidance and training; and (4) launch pilot projects.

**Figure 1:**
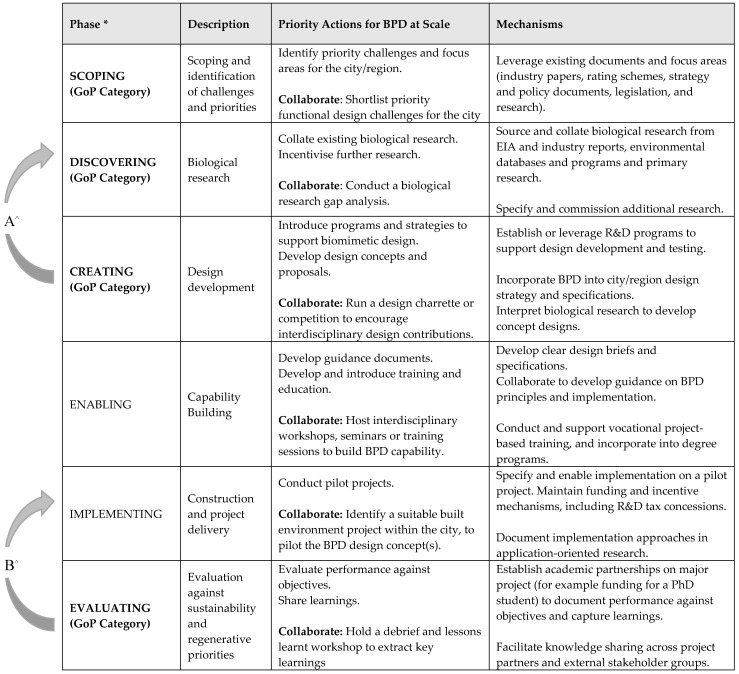
Priority capability-building actions for industry, government, and academia sectors to advance biomimetic place-based design (BPD) at-scale. * Bolded text indicates original GoP phases [8]. ^^^ Iterative linkages: A = During and after the ‘Creating’ phase, it may become evident that further investigation of the biological sources is required. B = Evaluation should occur both before and after implementation to support the identification of gaps and opportunities for improvement.

While participants were not asked directly to comment on actions around scoping and evaluation phases, these emerged in the workshops’ outputs and have been reflected in the discussion and Figure 1 below. Recognising the role of many and varied influencers in complex systems innovation, participants were also asked to consider who would be best placed to lead each action, with three key sectors proposed: (1) industry; (2) government; and (3) academia.

### 3.3. Workshop Analysis

Following each of the three workshops, raw data captured on worksheets were collated and transcribed into Excel for further analysis. Initial analysis included the revision of responses from each small working group to the BPD activity. Following this preliminary review, responses were collated first into summaries for each workshop and reviewed for key emerging themes and concepts. As the first workshop was targeted as a scoping workshop, its results were maintained as standalone findings. For the Australian workshops, recognising their common objectives and similar participant profiles, activity responses from the Brisbane and Sydney workshops were then merged to create a summary of findings and emergent concepts.

Results were reviewed to identify key actions and priority areas, with duplicates removed and consolidated. Following this, thematic analysis was conducted on emergent clusters of priorities and actions, highlighting five key themes common across the two workshops, as outlined in Section 4.3. Priority actions were coded against these themes, as well as the sectors they had been attributed to, before internal review and validation was conducted by the first two authors and one workshop facilitator. Findings were synthesised in Excel worksheets, and several mapping and coding initiatives were undertaken, including consideration of priority actions relative to emergent themes (information and analysis; network and knowledge sharing; funding and incentives; scoping, and frameworks and standards). The workshop findings were also mapped against the existing Genius of Place design methodology, including the four phases of scoping, discovering, creating and evaluating, to provide a structured scaffold, and to highlight where actions and challenges proposed by workshop participants diverged from the existing design methodology.

## 4. Results

The following paragraphs summarise results from the three workshops, which are then used as the foundation for an action framework to support BPD moving forward, as well as providing insights into the potential roles for each sector.

### 4.1. Workshop 1: USA Scoping Workshop (Academic)

In the preliminary scoping workshop, participants identified several key opportunities associated with the BPD approach, including the GoP methodology. These included opportunities to learn from ‘champion adapters’ in different regions and countries facing similar challenges and operating conditions; the opportunity for context-specific and place-based design—“Value of context-specific opportunities” (WS1-WG1)—and a shift towards a comprehensive, rather than individualistic, understanding of place, “Moving from individual aspects to comprehensive review of a place” (WS1-WG1). Further, participants noted an opportunity to “Look at extremophile, champion adapters, those that live in highly-disturbed environments” (WS1-WG3) as another lens and source of inspiration. Challenges identified included the increasingly degraded and impacted nature of reference ecosystems; the need for biology expertise; and challenges associated with reconciling the social, ecological, cultural, and industrial contexts as they relate to the built environment design.

### 4.2. Workshop 2 and 3: Australian Engagement Workshops (Industry)

The industry workshop participants were provided with additional framing for the BPD activity, with four key phases (biological research; develop design; develop guidance and training; launch pilot projects) and three key sectors (industry, academia, and government) used to structure the workshop responses. Further, activities elicited specific actions and opportunities for advancing BPD at the city scale. In combination, this structuring generated a number of priority action areas for each sector across each phase of the BPD cycle. The following paragraphs summarise key actions identified at each phase.

#### 4.2.1. Biological Research

Participants identified a role for industry in identifying research needs (“Conduct needs analysis” (WS3-WG1), and the provision of grant funding for biological research through PhD and Master’s’ research scholarships, R&D funding programs, and employee study opportunities. Looking at existing structures, they highlighted the role of industry associations in conducting research, as well as the opportunity to leverage environmental impact assessments to collate relevant biological information and data. Recognising the importance of interdisciplinary collaboration and communication, participants also noted the need to “Apply initiatives/opportunities from academia in industry sectors” (WS2-WG1&2). Opportunities for government to contribute to biological research included drawing on “local government knowledge of place” (WS2-WG1&2), as well as extending or supplementing existing governmental research agencies, datasets, and reporting frameworks (for example, historical and projected climate data, or leveraging work undertaken by environmental and infrastructure departments). Research could also be integrated with broader planning and strategy documentation, including through the identification of biological research focus areas. Scoping and provision of grants and funding, and incentives for industry development and uptake, were also highlighted as important roles for government. 

For academia, the completion of academic literature reviews and studies was deemed to be an important early deliverable, as well as case studies of existing applications, and consolidation of outcomes from available environmental impact assessments to contribute to the biological research. Those in academia are uniquely placed to progress the theory, methodologies, and underlying research requirements for BPD, including through research and development collaborations with industry and government partners. Finally, there is an opportunity to incorporate BPD into curriculum coursework and assessment, allowing for ongoing investigation and capability-building.

#### 4.2.2. Design Development

The industry sector is likely to play a leading role in design development, including through the generation of focused and specialised design strategies derived from biological insights for implementation, as well as the establishment of research and development programs within organisations. To support design development, participants recommended the provision of an overarching design strategy by the government, as well as updated specifications and regulations to support and catalyse further uptake. With the appropriate provision of funding, government agencies would collaborate with industry to ensure that design approaches were aligned with government policy—“Work with industry when developing design strategies by giving context and outline on what is required for policymaking, so that design strategies don’t end up clouding or negating government policy” (WS3-WG3). This also included supporting research outcomes to continuously inform legislation and guidelines—“Incorporate design strategies in updated specification and guidelines” (WS2). An important opportunity for academia lies in the potential to provide insight into current practice and a roadmap for progression, including, for example, a “biomimicry ‘audit’ of key city infrastructure” (WS2-WG1&2); interpretation of biological research and strategies to inform design; and opportunities to amend teaching curriculum to reflect biomimetic design approaches and strategies.

#### 4.2.3. Guidance and Training

Participants noted the importance of industry working with the government and researchers to develop and review guidance and training. They highlighted the importance of developing clear briefs to assist in guiding implementation and industry uptake, as well as delivery of training courses and “vocational and project-based training” (WS3-WG2) in biomimetic place-based designs. Industry associations were highlighted for their role in encouraging innovation (“Industry associations like ISCA encourage biomimicry as innovation” (WS2-WG1&2)), with industry facilitating a non-competitive ideas and guidance group to publish common guidance materials. The role for government in developing guidance and training was significant, with the opportunity to engage specialists, consultants, academics and industry bodies to collaboratively develop place-based guidelines and design strategies for industry, including “publishing design guidelines for different places” (WS3-WG3). Similarly, the government could support research and development through the establishment of applied research programs, drivers for inclusion within education programs, and the trial and testing of new materials on government projects. Access to academic research would be integral to the development of guidance on principles and implementation, with a focus on ensuring that assessment outputs are shared directly with government and industry sectors. Participants highlighted the importance of academia working with industry to ensure that such content is practical, as well as the opportunity for integrating biomimicry introductory content into university courses.

#### 4.2.4. Pilot Projects

In pilot projects, industry plays a primary role in implementing the research and guidance of the above phases, as well as sharing outcomes with industry and government. Participants again noted the role of industry associations, who may contribute to promoting, supporting, and sharing learnings from pilot projects. The provision of incentives and funding for pilot projects was a key opportunity for the government in this phase, supported by the government’s role in shaping specifications and contract requirements to support piloting. Participants noted the opportunity for the government to proactively encourage pilot projects without waiting for comprehensive market demand—“Facilitate implementation on a pilot project—spec and enable” (WS3-WG3), and suggested mandated knowledge sharing provisions within the project contracts to support broader learning. Tax relief and R&D funding mechanisms could be leveraged to support early pilots and testing, as well as “government-funded academic partnerships” (WS2-WG1&2)—for example a PhD candidate embedded within the project team for a major project—to ensure that performance and learnings are captured and evaluated.

Finally, academia would play an important role in the assessment of project outputs against established goals and targets. Researchers could add value to pilot projects through the testing of pain points (with insight from industry) to inform further research and knowledge sharing. This collaborative approach would support meaningful research while distributing the cost burden between industry and academia.

### 4.3. Emergent Themes and Priority Action Areas

The above findings describe clear actions identified for industry, government, and academia sectors to support capability building for BPD and facilitate the adoption of this design approach at-scale. Stepping above the individual phases and analysing these actions as an aggregated group, several key themes and priority areas emerged, as summarised in Table 2. These themes reflect the overarching categories of capability-building actions identified by workshop participants and include (1) information and analysis; (2) network and knowledge sharing; (3) funding and incentives; (4) scoping; and (5) frameworks and standards. These themes group the workshop results into overarching concepts for prioritized focus moving forward. Table 2 illustrates the number of actions coded against each theme, including the sector that participants identified as best placed to lead those efforts. It offers a high-level heat map of the workshop outcomes. Key results are summarized underneath Table 2. 

Table 2 illustrates important roles for the industry, government, and academia sectors in enabling BPD at-scale. There was a clear reliance on the government for information, funding, and incentives to build capability, as well as a perceived role for government in crafting and scoping of specifications and contract requirements—reflecting the fact that the phrasing of such clauses can impact not only the extent to which innovative initiatives may be implemented, but whether they are even possible at all. 

The results indicate a reliance on academia to support capability building through information provision and robust analysis, as well as by leveraging existing education frameworks and standards. The importance of early and rigorous research and evaluation was a recurring theme. Participants also noted that academic investigation must be integrated with government and industry practices in order to produce meaningful results. For industry, in addition to a leading role in pilot project implementation, results highlighted an expectation that the industry sector should lead and facilitate capability building through strong networks and knowledge sharing.

### 4.4. An Action Framework for Advancing Biomimetic Place-Based Design

Synthesising the actions across each phase, Figure 1 proposes priority actions for scaling BPD moving forward, using the GoP 4 phases [8] as a framework for structuring these actions and priority areas. Where the GoP methodology offers a detailed process for translating biology to design at the project level, Figure 1 offers a higher-level consideration of how the necessary foundations, resources, and capabilities for BPD may be further developed at a city-level. Extensive work has already been undertaken to develop and refine the GoP design methodology (See Baumeister et al., 2013), and as such the focus here is to supplement that existing framework by identifying the additional elements that become important when shifting focus from project to city or region-level application.

In exploring how BPD may apply at-scale, Figure 1 highlights opportunities for industry, government, and academia to advance the BPD approach by leveraging and expanding existing structures, frameworks, and information sources. Drawing on the GoP 4 phases (Column 1), the table supplements these with the inclusion of new ‘enabling’ and ‘implementing’ phases, which are of particular relevance when considering capability building and implementation at-scale. Column 3 presents high-level actions for leveraging and building capability, while Column 4 suggests mechanisms for doing so. 

## 5. Discussion

In the following paragraphs, the authors discuss the proposed BPD phases and framework, which together address key challenges and barriers to scaling BPD by enabling streamlining and capability building.

### Capability Building for BPD at Scale

In Figure 1, the GoP phases proposed by Baumeister et al. [8] are supplemented with two additional phases, recognising the different priorities and challenges of applying biomimetic design at-scale. ‘Enabling’—focused on building capability, and ‘Implementing’—focused on piloting and applying the proposed design approaches in practice in order to test, evaluate, and learn. These are designed to support the scaling of the GoP approach. We now discuss Figure 1 in the context of the proposed BPD phases.

Scoping: key actions identified for the scoping phase include drawing on existing industry reports, as well as planning and policy documentation and research agendas to identify priority challenges faced by the industry, government, and academia sectors in that city or region. While not a perfect proxy for local design challenges, this approach will generate a list of key challenges faced by the local area in terms of environmental, social, political, and economic considerations and interrelationships—an insight into the local ‘operating conditions’, hurdles and priorities. This could include, as an example, identifying that flooding is a major concern for the city, and must be a priority consideration in design moving forward. The design challenge(s) would then be articulated in terms of function, i.e., ‘regulate hydrological flows’, ‘divert water’, ‘slow and purify water’, and ‘manage erosion and sediment control’, and relevant design priorities and principles could be established on that basis.

Discovering: In the discovering phase, the objective is to biologize the design question or challenge, translate natural models and abstract biological strategies into design principles. In the above example, a local mangrove species may provide an interesting and relevant model for regulating hydrological flows or calming incoming water. Once natural models are identified, it is then possible to investigate in more detail the specific strategies and design principles that the organism uses to achieve the functional outcome. To support this phase, it will be necessary to understand, at the city-level, what biological and ecosystem information is currently available, and what research gaps remain. Biological and ecological research conducted for environmental impact assessments, for example, can be drawn on as a foundation insight into key local species and ecosystem challenges. Further to this, many open-source databases may be readily accessed to support place-based biological research and data gathering, including: Ask Nature [40], iNaturalist [41], and eBird [42], as well as the United Nations Integrated Biodiversity Assessment Tool [43]. Other frameworks can also be used for identifying focus species for a selected location. Work by Apfelbeck et al. [44], for example, offers a framework for selecting target species for wildlife-inclusive urban design that takes into account local species, habitat availability, and site suitability, and could be readily adapted for use in biomimetic place-based design. For the government sector, actions included leveraging comprehensive existing environmental datasets, developing specifications to guide research, and encouraging further biological research through direct commissioning, funding, and incentives. Academia will play an important role in sourcing and collating primary ecological data, as well as conducting additional research into individual species, strategies, and adaptations.

Creating: In the ‘Creating’ phase, design ideas are brainstormed and design principles emulated. Here, the industry sector can support innovative design work through research and development programs, as well as through direct efforts to develop and pilot concept designs. The government can ensure that the biological research conducted in the ‘discovering’ phase is reflected in government guidance and specifications through the development of an overarching design strategy and a commitment to actively integrate biomimetic design requirements into specifications. Academic specialist input will be integral in the translation of biological strategies into built environment design approaches, as well as the ongoing generation of biomimetic design concepts through higher education programs.

Enabling: The enabling phase (included here in addition to the 4 phases of the GoP biomimetic design approach) focuses on BPD capability building through the development of guidance and training. Recognising that BPD concepts remain relatively new across each sector, actions included cross-sectoral collaboration to draft BPD best practice guidance and develop vocational project-based training, as well as a commitment to developing clear briefs and specifications for the supply chain. For government, opportunities included building on existing design concepts and requirements (such as water sensitive urban design, for example) to integrate BPD. For academia, proactive sharing of research and evaluation findings, as well as the incorporation of BPD into undergraduate and continuing professional development programs were identified as key priorities.

Implementing: As per the enabling phase, the ‘implementing’ phase was inserted in addition to the 4 phases of the GoP methodology to capture key actions required for piloting and ongoing implementation of biomimetic place-based design approaches. Here, industry commitment to undertaking pilot projects could be supported by the government specifying and incentivizing implementation, including maintaining or expanding funding mechanisms. The role of academia includes the generation of industry-focused research to inform pilot projects, in addition to the documentation of implementation approaches to support future refinement and uptake.

Evaluating: Robust evaluation will be integral to the continued uptake and development of a city-wide BPD effort. For the industry sector, this would involve documentation and sharing of learnings from applied projects, and for the Government sector, the creation of academic partnerships on major projects to capture learnings and advance the knowledge base. For academia, rigorous assessment of project outcomes relative to goals and objectives, and investigation of priority pain points on early projects will be integral to informing approaches for future projects.

## 6. Conclusions and Future Directions

Biomimicry offers an opportunity to not only mitigate risk to local ecosystems, but to directly draw on them for inspiration and design guidance—shifting from narrow damage control towards regenerative design by drawing on locally-attuned design strategies that are adaptive, resilient, and multi-functional. This paper has presented a preliminary framework for increasing the capability of industry, government, and academia sectors to implement a biomimetic place-based design at-scale. While the GoP design methodology offers an established process for BPD at the project level, this paper supplements the existing process with key considerations for the advancement of this concept at the city-level.

Reflecting the importance of ‘learning through doing’, an important first step will be the piloting of city-level BPD studies to validate benefits and barriers to implementation. This work could begin with a gap analysis of data availability and biological research requirements for applying BPD approaches in a selected city. Once piloted, government agencies have an opportunity to incorporate BPD concepts and design approaches into city-level priorities and strategic planning. This would catalyse broader research and implementation across various sectors and design contexts. Through collaboratively leveraging and building on existing practices, there is a tangible opportunity for the industry, government, and academia sectors to introduce BPD approaches at-scale in support of resilience and regenerative design. This framework offers a resource to guide and support such efforts.

## Figures and Tables

**Table 1:** BPD workshops: meta-data summary.

Investigation Phase	Workshop	Code	Location	Academic/Industry Participants	Total Participants	Working Groups
Scoping	Workshop 1 7 August 2018	WS1	Phoenix, Arizona	13/2	**15**	WG1 WG2 WG3
Industry Engagement	Workshop 2 5 February 2019	WS2	Brisbane, Australia	0/9	**9**	WG1&2 *
	Workshop 3 8 February 2019	WS3	Sydney, Australia	1/14	**15**	WG1 WG2 WG3

* The Brisbane workshop involved a common whiteboard for capturing/displaying contributions.

**Table 2:** Coding of priority capability-building actions against emergent themes and sectors.

Capability-Building Actions	Sector (I/G/A)	Actions Allocated (#)	Total Actions (#)
Information and analysis	I	3	19
	G	7	
	A	9	
Network and knowledge sharing	I	6	12
	G	3	
	A	3	
Funding and incentives	I	2	9
	G	7	
	A	0	
Scoping	I	2	12
	G	10	
	A	0	
Frameworks and standards	I	3	11
	G	4	
	A	4	
**Totals:**	I = 16; G = 31; A = 16; ALL = 63		

Legend: I = Industry; G = Government; A = Academia. Shading depicts number of actions allocated (darker = more actions).
